# Impact of Sarcopenia on Prognosis, Treatment Toxicity and Surgical Complications in Locally Advanced Gastric Cancer

**DOI:** 10.3390/cancers18091430

**Published:** 2026-04-30

**Authors:** David da Silva Dias, Paulo Luz, Ana Fortuna, Ana Águas, Mafalda Machado, Beatriz Gosálbez, Rosa Farate, Rita Clemente Pinho, Ana Carmo Valente, José Leão Mendes, Marta Maria Seladas, Carolina Trabulo, Paula Ravasco

**Affiliations:** 1 Medical Oncology Department, Unidade Local de Saúde Cova da Beira, 6200 Covilhã, Portugal; 2 Faculty of Health Sciences, Universidade da Beira Interior (FCS-UBI), 6200 Covilhã, Portugal; 3 Clinical Academic Center of Beiras (CACB), 6200 Covilhã, Portugal; 4 Center for Interdisciplinary Research in Health (CIIS), Universidade Católica Portuguesa, 1649 Lisbon, Portugal; 5 Medical Oncology Department, Unidade Local de Saúde Algarve, 8000 Faro, Portugalana.fortuna@ulssm.min-saude.pt (A.F.);; 6 Radiology Department, Unidade Local de Saúde Cova da Beira, 6200 Covilhã, Portugal; 7 Medical Oncology Department, Unidade Local de Saúde de São João, 4200 Porto, Portugal; 8 Medical Oncology Department, Unidade Local de Saúde São José, 1169 Lisbon, Portugal; 9 Clinical Academic Center Lisbon (CCAL), 1649 Lisboa, Portugal; 10 Medical Oncology Department, Unidade Local de Saúde Arco Ribeirinho, 2834 Barreiro, Portugal; 11 Faculty of Medicine, Universidade Católica Portuguesa, 2635 Rio de Mouro, Portugal; 12 Egas Moniz Center for Interdisciplinary Research, Instituto Universitário Egas Moniz, 2829 Almada, Portugal

**Keywords:** gastric cancer, skeletal muscle mass, body composition, sarcopenia, dose-limiting toxicities, postoperative complications

## Abstract

**Background:** Weight loss and skeletal muscle wasting are frequent in cancer and may influence treatment tolerance and outcomes. Computed tomography (CT) based body composition analysis at the third lumbar vertebra (L3) is an accurate method to quantify skeletal muscle in routine oncology care. **Methods:** We performed a multicenter retrospective cohort study including 202 adults with locally advanced (stage IB–III) gastric cancer treated in four Portuguese hospitals (January 2020–December 2022). Skeletal muscle area (SMA) was assessed on baseline CT at the L3 vertebral level, using Data Analysis Facilitation Suite (DAFS) software v3.11.2, and skeletal muscle index (SMI) was subsequently calculated. Patients with low muscle quantity were classified as sarcopenic (below sex-specific SMI mean). We evaluated associations with relapse-free survival (RFS), overall survival (OS), FLOT chemotherapy dose-limiting toxicities (DLTs), and postoperative complications after gastrectomy. **Results:** Mean age was 69 years, 65% had ECOG PS 0, 53% received FLOT chemotherapy protocol. Mean SMI was 49.6 cm^2^/m^2^ in males and 40.9 cm^2^/m^2^ in females and correlated positively, though moderately, with BMI (*p* < 0.01; r = 0.424). Sarcopenia was not significantly associated with RFS (*p* = 0.186) or OS (*p* = 0.168) at 30-month follow-up. Although numerical differences were observed (64% vs. 56% of patients did not relapse and 74% vs. 63% were alive, for non-sarcopenic vs. sarcopenic patients). Sarcopenia was associated with a higher risk of DLTs (*p* = 0.021; OR 2.56, 95% CI 1.15–5.73) and postoperative complications (*p* = 0.024; OR 2.16, 95% CI 1.11–4.21). **Conclusions:** Sarcopenia significantly increases the risk of chemotherapy toxicity and postoperative complications in locally advanced gastric cancer. However, its effect on OS and RFS was not statistically significant at 30-month follow-up. Standardization of CT-based sarcopenia cut-offs remains a major barrier to clinical implementation.

## Simple Summary

Multicenter retrospective cohort study, with 202 adults with locally advanced stage (IB-III) gastric cancer treated in four Portuguese hospitals between January 2020 and December 2022. Baseline CT was used to measure skeletal muscle quantity at L3 vertebral level. Patients with low muscle quantity were classified as sarcopenic and presented increased risk of ChT dose-limiting toxicities and postoperative complications. They presented a tendency to lower overall survival and relapse-free survival, although this was not statistically significant. Standardization of cut-offs defining sarcopenia by CT scan remains a major barrier to clinical implementation.

## 1. Introduction

Gastric cancer (GC) is the fifth most frequently diagnosed cancer in Portugal, with 3668 new cases reported in 2022, and the third leading cause of cancer-related death, accounting for approximately 2578 deaths [1].

The incidence is more prevalent in males, although has significant variations according to geographic locations. High rates are observed in Eastern Asia, Central and Eastern Europe and South America, and lower rates are observed in North America and Western Europe [2]. The mortality rates are higher in North America and Europe than in Asia, probably because there is no widespread screening for gastric cancer in these countries [3]. New molecular targets and therapeutics bring new hope for the treatment of GC.

Since 2019, perioperative chemotherapy (ChT) FLOT protocol was used for the treatment of locally advanced gastric cancer (LA-GC), including stages IB–IIIC. Perioperative protocol is used in 14-day cycles, four cycles before surgery and four cycles after surgery, including intravenous agents (fluorouracil-5-FU 2600 mg/m^2^, leucovorin 200 mg/m^2^, oxaliplatin 85 mg/m^2^, docetaxel 50 mg/m^2^). The median overall survival (OS) at the 5-year follow-up was 50 months. After 5 years, 45% of patients were alive [4].

Body composition, including skeletal muscle mass, can be evaluated by several methods such as anthropometric measurements, bioelectrical impedance analysis (BIA), dual-energy X-ray absorptiometry (DXA), computed tomography (CT), and magnetic resonance imaging (MRI) [5]. CT assessment of body composition at L3 vertebral level is as precise as DXA assessment, and it is routinely performed at diagnosis and follow-up in cancer patients, making it a practical method to use [6].

Alteration of body composition in cancer patients, including weight loss and especially loss of skeletal muscle, also known as sarcopenia, is common and clinically relevant. These alterations occur due to multifactorial mechanisms such as reduced intake, systemic inflammation, metabolic dysregulation and treatment-related effects [7].

The term sarcopenia originates from the Greek words sarx (flesh) and penia (loss) and is recognized as a disease in the ICD-10-CM classification (M62.83) [8].

According to the European Working Group on Sarcopenia in Older People (EWGSOP), it is defined as a progressive and generalized skeletal disorder associated with an increased likelihood of adverse outcomes including falls, fractures, physical disability and mortality. The diagnosis is probable if low muscle strength is present, but confirmation requires low muscle quantity or quality [9].

Sarcopenia, assessed by CT scan at L3 vertebra, provides important data across several tumors, including worse outcomes, higher chemotherapy toxicity, and postoperative complications across cancer types, including GC [10]. Implementation into clinical practice is limited by heterogeneity in CT-based definitions and cut-offs. These values often vary according to region, ethnicity, tumor type, and treatment [7,11].

The main objective of this study is to evaluate the impact of sarcopenia on DLTs during FLOT ChT before surgery, in LA-GC in a Portuguese population. Secondary objectives included the evaluation of the impact of sarcopenia on OS, RFS and postoperative complications.

## 2. Materials and Methods

### 2.1. Patients and Procedures

This retrospective multicenter cohort study included patients treated between January 2020 and December 2022 in four Portuguese hospital institutions. Adult patients (≥18 years) were eligible if they had histologically confirmed gastric adenocarcinoma stage IB-III. Locally advanced disease (non-metastatic) was defined as tumors extending beyond the early stage (stage IA), involving deeper layers of gastric wall and/or regional lymph nodes, but without evidence of distant metastases. This definition is consistent with the eligibility criteria of the FLOT4-AIO trial.

All patients were required to have a baseline CT scan available for body composition analysis. Baseline CT was defined as a CT scan performed within 8 weeks before surgery or the start of preoperative ChT. Exclusion criteria included synchronous tumors, evidence of metastatic disease at baseline, previous ChT, unavailable CT images and loss to follow-up. 

This study was conducted in accordance with the principles of the declaration of Helsinki and was approved by the ethics committee of all four participating institutions. Data were collected from electronic medical records and included demographic variables (sex, age, ECOG PS), as well as anthropometric measurements (body weight and height). Tumor and treatment variables were also recorded, including histological subtype, postoperative complications and treatment-related toxicities, which were graded according to the common terminology criteria of adverse events—CTCEA v6.0.

**Figure 1:**
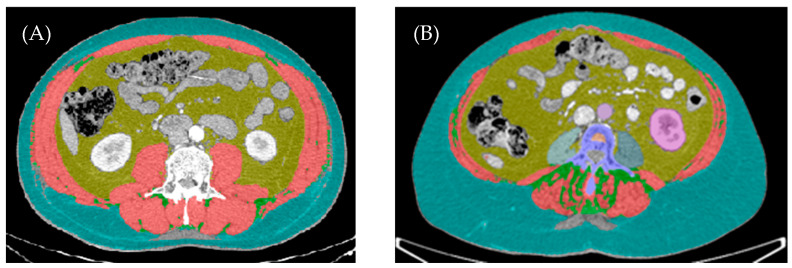
CT scan assessment at the L3 vertebral using DAFS software. Red—skeletal muscle area. Yellow—visceral adipose tissue area. Blue—subcutaneous adipose tissue area. Green—intramuscular adipose tissue area. Representation of 2 patients: (**A**) male, overweight (BMI 26.7 kg/m^2^) without sarcopenia/high muscle quantity (SMI 67.8 cm^2^/m^2^), (**B**) female, obese (BMI 34.8 kg/m^2^) with sarcopenia (SMI 36.8 cm^2^/m^2^).

Baseline CT scans were analyzed at the L3 vertebral level using DAFS software v3.11.2 (https://www.voronoihealthanalytics.com/dafs accessed on 26 April 2026), to quantify muscle quantity. Each CT scan was reviewed and subjected to quality control by an independent radiologist. The following muscles were included in the analysis: quadratus lumborum, latissimus dorsi, psoas major, erector spinae, abdominal oblique muscles, and rectus abdominal. Skeletal muscle area (SMA) was quantified using a predefined attenuation range of −29 to 150 Hounsfield units (HU) and expressed in square centimeters (cm^2^). Skeletal muscle index (SMI) was calculated by normalizing SMA to height squared and is reported as cm^2^/m^2^. SMI was calculated for the male and female groups. For this analysis, sarcopenia was defined as SMI below the sex-specific cohort mean. Figure 1 illustrates an example of body composition assessment, using DAFS software, to analyze CT images at the L3 vertebral level in two patients with LA-GC.

### 2.2. Outcomes

The primary objective was to assess the impact of sarcopenia on dose-limiting toxicities (DLTs) during the four preoperative cycles of perioperative FLOT ChT. DLTs were defined as treatment-related toxicities requiring ChT dose reduction, treatment delay or discontinuation. The secondary endpoints were the impact of sarcopenia on postoperative complications after gastrectomy (complications were defined as relevant if Clavien–Dindo grade ≥ 2), and the impact of sarcopenia on OS and RFS.

### 2.3. Statistical Analysis

Statistical analyses were conducted using IBM SPSS Statistics version 30. Missing data were handled using listwise deletion method. Continuous variables are presented as means ± standard deviation. For normally distributed variables, differences between groups were assessed using the independent samples *t*-test. Associations between categorical variables were evaluated using chi square test. Correlation between SMI and BMI was assessed using Pearson correlation coefficient. Survival analyses were performed using the *Kaplan–Meier* method and compared with the *log-rank* test. Logistic regression and Cox proportional hazards models were also applied. Results were reported as odds ratio (OR) and hazard ratio (HR), with corresponding 95% confidence intervals. Statistical significance was defined as *p* < 0.05.

## 3. Results

### 3.1. Baseline Characteristics

A total of 202 patients with LA-GC were included. Mean age was 69 years, and most patients (65.3%) had an ECOG performance status of 0. Regarding tumor characteristics, disease stage was an important factor in this analysis: 50 patients had stage IB disease, 72 had stage II and 80 had stage III. All cases were gastric adenocarcinoma, with the intestinal subtype being the most common (61.4%). Histopathological analysis of surgical specimens showed vascular, lymphatic and perineural invasion in 55%, 32% and 50% of patients, respectively. In terms of treatment, nearly all patients underwent surgery. Perioperative ChT with the FLOT regimen was administered to 106 patients, of whom 97 initiated treatments at full dose. Detailed data are presented in Table 1.

**Figure 2:**
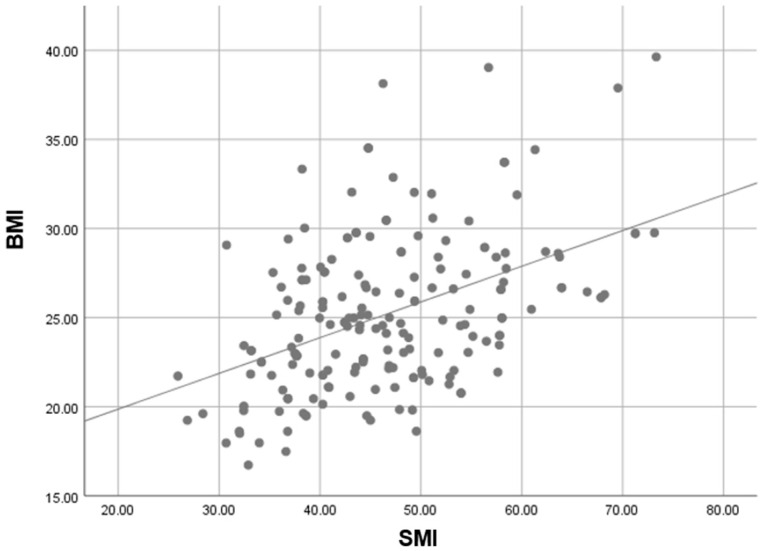
Correlation between BMI and SMI. A positive but moderate correlation was observed, indicating that low muscle mass may occur across all BMI categories.

According to body composition, most patients were of normal weight (51.8%). The mean SMI was 49.6 cm^2^/m^2^ in males and 40.9 cm^2^/m^2^ in females. SMI showed a moderate positive correlation with BMI (*p* < 0.01; r = 0.424), indicating that higher BMI is generally associated with higher muscle mass. However, the strength of this correlation suggests that low muscle mass may occur across all BMI categories, including in overweight patients, highlighting the limitations of BMI as a surrogate for body composition (Figure 2).

### 3.2. Dose-Limiting Toxicities

From the 202 patients, 106 underwent perioperative ChT with the FLOT protocol. Some patients were considered unfit for FLOT and received alternative preoperative regimens, such as FOLFOX (fluorouracil, leucovorin, and oxaliplatin), while others proceeded directly to surgery. The main reasons included gastrointestinal obstruction, tumor-related bleeding, and reasons not specified in the electronic medical records.

The most frequent severe toxicities among patients who underwent perioperative ChT with FLOT regimen included neutropenia, diarrhea, nausea and peripheral neuropathy. Treatment was delayed in 35 patients (34%), dose reduction was required in 33 (32.1%), and 4 (3.8%) discontinued treatment due to toxicity. Overall, 45 patients (42.5%) experienced DLTs, defined as treatment-related toxicities requiring ChT dose reduction, treatment delay or discontinuation. 

Out of the 106 patients treated with FLOT, 97 initiated therapy with full dose (100%). To evaluate the impact on DLTs, only patients who started treatment at full dose were included in the analysis.

Sarcopenia was the only factor significantly associated with an increased risk of DLTs (*p* = 0.021; OR 2.56; 95% CI 1.15–5.73), as shown in Table 2.

### 3.3. Postoperative Complications

Clinically relevant postoperative complications were observed in 63 (32.6%) patients, assessed as Clavien–Dindo grade ≥2. Patients with grade 2 were 36 (18.7%), grade 3 were 21 (10.9%) and grade 4 were 6 (3.1%). Out of the six patients with grade 4 complications requiring ICU support, three patients died in the 30 days after surgery. The most common complications included anastomotic leak and infectious events such as surgical site infection, pneumonia, peritonitis and intra-abdominal abscess.

ECOG PS ≥ 2 (*p*= 0.033; OR 1.68; 95% CI 1.04–2.71) and Sarcopenia (*p* = 0.024; OR 2.16; 95% CI 1.11–4.21) were associated with higher odds of postoperative complications as represented in Table 3.

### 3.4. Overall Survival and Relapse-Free Survival

**Figure 3:**
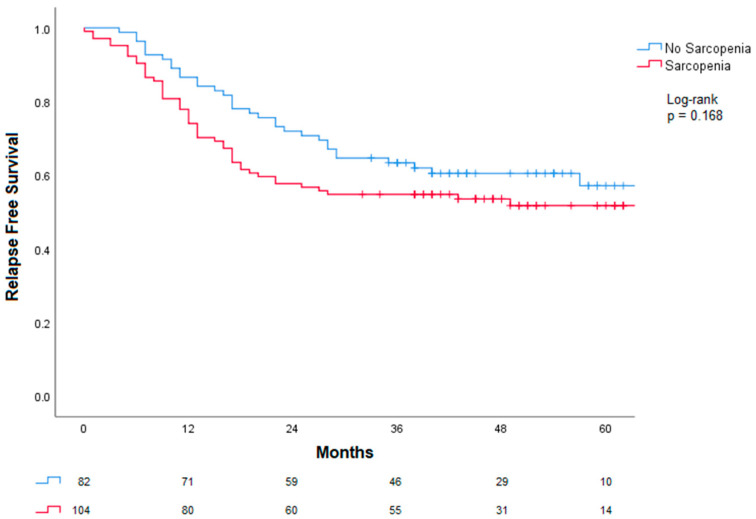
Association between sarcopenia and relapse-free survival. A numerical difference was observed between groups; however, this did not reach statistical significance.

Regarding relapse-free survival, after 30 months, sarcopenia was not associated with RFS (Figure 3). A numerical difference was observed: 64% of patients without sarcopenia were disease-free versus 56% with sarcopenia; however, this difference was not statistically significant (log-rank *p* = 0.186).

**Figure 4:**
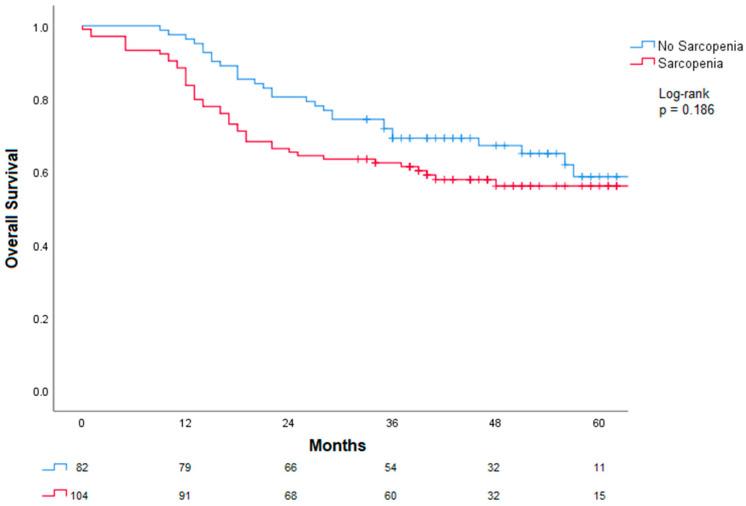
Association between sarcopenia and overall survival. A numerical difference was observed between groups; however, this did not reach statistical significance.

Regarding overall survival, after the 30-month follow-up, sarcopenia was not associated with OS (Figure 4). A numerical difference was observed: 74% of patients without sarcopenia were alive versus 63% with sarcopenia; however, this difference was not statistically significant (log-rank *p* = 0.168).

When analyzing other factors influencing overall survival, disease stage, lymphatic, vascular and perineural invasion, were associated with worst prognosis in a univariate Cox regression analysis. Patients who completed all eight cycles of ChT with FLOT protocol had better prognosis compared with those who did not. In a multivariable Cox regression model, tumor stage remained the only independent predictor of overall survival (*p* = 0.001; HR 5.02, 95% CI 2.02–12.48). Further details are presented in Table 4.

**Figure 5:**
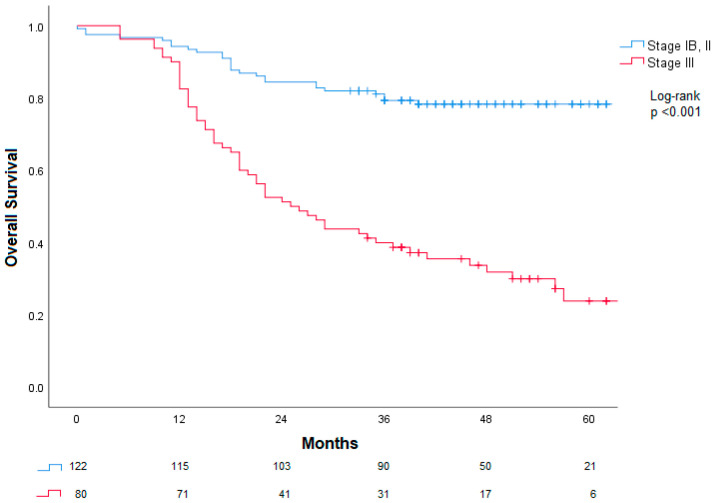
Association between disease stage and overall survival. Stage III disease had significantly worse overall survival compared with stages IB and II.

Considering disease stage, patients with stage III had significantly worse prognosis compared to those with stage IB and II, as shown in Figure 5 (log-rank *p* < 0.001).

## 4. Discussion

Assessing body composition using CT provides important information with impact on clinical practice, such as chemotherapy toxicity, surgical complications, and prognosis. However, manual segmentation of CT images is labor intensive and can lead to significant interobserver variability. For this reason, DAFS software was used in this study as it enables rapid and accurate body composition analysis by automating segmentation, reducing expert workload and computational burden, especially in large sample studies [12].

Although it can be assessed at different cross-section levels, it is usually measured at the level of the L3 vertebra, as there is a close correlation between muscle and fat tissue areas and corresponding tissue volumes; therefore, it is considered a reference point for body composition assessment in both sexes [6,13].

In this study, a median overall survival was not reached at the 30-month follow-up. Due to the limited number of survival events in this period, ROC analysis was not performed to calculate optimal SMI cut-off value for this population. Therefore, the mean SMI was used to differentiate patients with higher and lower muscle quantity, in this study characterized as sarcopenic patients and non-sarcopenic patients. The mean SMI in this population of LA-GA was 49.6 cm^2^/m^2^ in males and 40.9 cm^2^/m^2^ in females. Establishing a definitive cut-off value for sarcopenia is challenging, as thresholds vary according to different regions, cancer types and tumor staging. A cut-off that is frequently used in the literature is SMI < 52.40 cm^2^/m^2^ for men and <38.50 cm^2^/m^2^ for women, as seen in a Canadian study with several tumor types [14]. In contrast, Asian populations usually present lower cut-off values, such as <36 cm^2^/m^2^ for men and <29 cm^2^/m^2^ for women [15]. As an example, in a Portuguese study of advanced non-small cell lung carcinoma patients, sarcopenia cut-offs were <49.96 cm^2^/m^2^ for men and <34.02 cm^2^/m^2^ for women. A sub-analysis was performed using the Canadian study cut-offs, resulting in 20% of patients being reclassified as non-sarcopenic patients, and no impact of sarcopenia on OS was observed (17.9 months vs. 20.11 months, *p* = 0.588), while the cohort specific sarcopenia thresholds were prognostic (12.75 months vs. 21.13 months; HR 1.654; 95% CI 1.20–2.29; *p* = 0.002) [11]. The authors therefore emphasize the importance of reaching a consensus among the major sarcopenia societies regarding the CT-based muscle quantity threshold for sarcopenia. This standardization would improve comparability across studies, strengthening scientific evidence, and facilitating the integration of sarcopenia assessment into clinical practice.

In this study, the main objective was to evaluate the impact of sarcopenia on DLTs in LA-GC. A 2.5-fold increased risk was observed in sarcopenic patients. These results are consistent with the literature [16,17,18]. From a clinical practice perspective, it provides valuable information to oncologists, as they can identify patients with higher probability of toxicities due to FLOT ChT, and start the first cycle with a lower dose and escalate if tolerated or implement closer monitoring during the treatment cycle. A structured telemonitoring protocol, involving the nursing team, could help detect early signs of toxicity, and reinforce supportive care measures. 

Secondary objectives included the impact of sarcopenia in postoperative complications, and survival outcomes. In this cohort, patients with sarcopenia presented a 2-fold increased risk of postoperative complications. These findings are also consistent with the literature [19,20,21].

These data are important to clinical practice as these patients with increased vulnerability may benefit from prehabilitation programs to improve muscle strength, muscle mass, physical condition and potentially reduce postoperative complications [22,23,24].

In this cohort, patients with sarcopenia tended to have worse prognosis in LA-GC, although this was not statistically significant. Several factors may explain these findings, such as data not yet matured, with 30-month follow-up, sample size or smaller effect size observed on locally advanced disease compared with metastatic disease, as there is a factor (surgery), reversing the disease trajectory.

Much of the literature suggests that sarcopenia impacts overall survival and relapse-free survival [25,26]. A study by Zheng HL et al., including 781 patients with LA-GC, showed prognostic value of preoperative sarcopenia on OS and RFS at a 10-year follow-up (39.61% vs. 58.71% and 39.61% vs. 57.84%, respectively). Sarcopenia was an independent risk factor for 10-year OS (HR = 1.467; 95% CI 1.169–1.839) [27].

This study has several strengths, such as being a multicentric study in a specific Portuguese population with gastric cancer, in a locally advanced disease setting. However, several limitations should be acknowledged, such as that this is a retrospective study, and data was not primarily meant for research; missing data were present and treated accordingly; and as a multicentric study, different CT scanners and acquisition parameters were used across institutions. Nevertheless, all images were in DICOM format, and were analyzed using DAFS software, with the generated outputs undergoing manual quality control by a radiologist. In addition, the use of the cohort SMI mean value allows for internal discrimination, but should not be used for external assessment.

Early screening of sarcopenia in cancer patients, including LA-GC patients, will help identify individuals in need of a personalized approach including pharmacological treatment, physical exercise, and nutrition intervention. These interventions are critical as we know the benefits of a well-established prehabilitation program, and the fact that Portuguese patients with LA-GC usually present, before surgery, insufficient mean caloric and protein intake, 20.6 Kcal/kg and 0.86 g/kg respectively, much lower than the recommendations of 25–30 Kcal/kg and 1.5 g/kg by ESPEN [28,29].

## 5. Conclusions

In a multicenter cohort of LA-GC, CT evaluation of sarcopenia was associated with an increased risk of dose-limiting toxicities to FLOT ChT and postoperative complications. OS and RFS were numerical but not statistically significant at the 30-month follow-up. Despite the clinical importance of these results, standardization of cut-offs defining sarcopenia remains a major barrier for scientific guidance and clinical implementation.

## Figures and Tables

**Table 1:** Baseline characteristics.

Variable	N = 202
Patient	
Age	69 [42–90]
Sex	
Male	127 [62.9%]
Female	75 [37.1%]
ECOG PS	
0	132 [65.3%]
1	56 [27.7%]
≥2	14 [7.0%]
Tumor	
Stage	
IB	50 [24.8%]
IIA	39 [19.3%]
IIB	33 [16.3%]
IIIA	35 [17.3%]
IIIB	37 [18.3%]
IIIC	8 [4%]
Histology	
Intestinal	124 [61.4%]
Diffuse	73 [36.1%]
Vascular invasion	54.9%
Lymphatic invasion	32.4%
Perineural invasion	49.5%
Treatment	
Surgery	193 [95.5%]
Chemotherapy with FLOT protocol	106 [52.5%]
Full dose FLOT (100%)	97/106 [91.1%]
Body composition	
BMI Group	
<18.5 kg/m^2^	5 [2.5%]
18.5–24.9 kg/m^2^	102 [51.8%]
25–29.9 kg/m^2^	70 [35.5%]
>30 kg/m^2^	20 [10.2%]
SMI mean (cm^2^/m^2^)	
Male [n = 119]	49.60 [±9.78]
Female [n = 67]	40.92 [±6.78]

**Table 2:** Factors influencing dose-limiting toxicities.

Variable	*p* Value	Odds Ratio (OR)
Age	*p* = 0.296	-
ECOG PS (≥2)	*p* = 0.062	-
Stage (I–II vs. III)	*p* = 0.344	-
Histology	*p* = 0.377	-
Lymphatic invasion	*p* = 0.193	-
Vascular invasion	*p* = 0.175	-
Perineural invasion	*p* = 0.333	-
BMI	*p* = 0.381	-
Sarcopenia	*p* = 0.021	OR 2.56; 95% CI 1.15–5.73

**Table 3:** Factors influencing postoperative complications.

	Univariate Logistic Regression		Multivariable Logistic Regression	
Variable	*p* Value	OR		OR
Age	*p* = 0.120	-	-	-
ECOG PS (≥2)	*p* = 0.015	OR 1.72; 95% CI 1.11–2.66	*p* = 0.033	OR 1.68; 95% CI 1.04–2.71
Stage (I–II vs. III)	*p* = 0.345	-	-	-
Histology	*p* = 0.480	-	-	-
Lymphatic invasion	*p* = 0.180	-	-	-
Vascular invasion	*p* = 0.408	-	-	-
Perineural invasion	*p* = 0.833	-	-	-
BMI	*p* = 0.389	-	-	-
Sarcopenia	*p* = 0.007	OR 2.53; 95% CI 1.77–4.71	*p* = 0.024	OR 2.16; 95% CI 1.11–4.21

**Table 4:** Factors influencing overall survival.

	Univariate Cox Regression		Multivariable Cox Regression	
Variable	*p* Value	HR	*p* Value	HR
Age	*p* = 0.751	-	-	-
ECOG PS (≥2)	*p* = 0.275	-	-	-
Stage (I–II vs. III)	*p* < 0.001	HR 4.57; 95% CI 2.86–7.31	*p* = 0.001	HR 5.02; 95% CI 2.02–12.48
Histology	*p* = 0.239	-	-	-
Lymphatic invasion	*p* = 0.012	HR 2.08; 95% CI 1.18–3.69	*p* = 0.147	-
Vascular invasion	*p* = 0.003	HR 2.05; 95% CI 1.27–3.29	*p* = 0.482	-
Perineural invasion	*p* < 0.001	HR 2.56; 95% CI 1.55–4.22	*p* = 0.072	-
Completed 8 cycles FLOT	*p* = 0.002	HR 0.38; 95% CI 0.22–0.67	*p* = 0.108	-
Sarcopenia	*p* = 0.173	-	-	-

## Data Availability

The original contributions presented in this study are included in the article. Further inquiries can be directed at the corresponding author.

## Abbreviations

The following abbreviations are used in this manuscript:

BIA	Bioimpedance analysis
BMI	Body Mass Index
CI	Confidence interval
ChT	Chemotherapy
CT	Computed tomography
CTCEA	Common Terminology Criteria for Adverse Events
DAFS	Data Analysis Facilitation Suite
DLTs	Dose-limiting toxicities
DXA	Dual-energy X-ray absorptiometry
EWGSOP	European Working Group on Sarcopenia in Older People
GC	Gastric cancer
HU	Hounsfield units
L3	Third Lumbar vertebra
LA-GC	Locally advanced gastric cancer
MRI	Magnetic resonance imaging
OS	Overall survival
OR	Odds ratio
PS	Performance status
RFS	Relapse-free survival
SMA	Skeletal muscle area
SMI	Skeletal muscle index
